# Supplementary material for: The Feasibility of Stereotactic Body Proton Beam Therapy for Pancreatic Cancer
Source: Cancers (Basel). 2022 Sep 20;14(19):4556. doi: 10.3390/cancers14194556 (PMC9559584; doi:10.3390/cancers14194556)
Supplement: Supplementary file 1 [file cancers-14-04556-s001.zip › cancers-1887510-supplementary.pdf]

Figure S1. Patient inclusion and exclusion criteria.

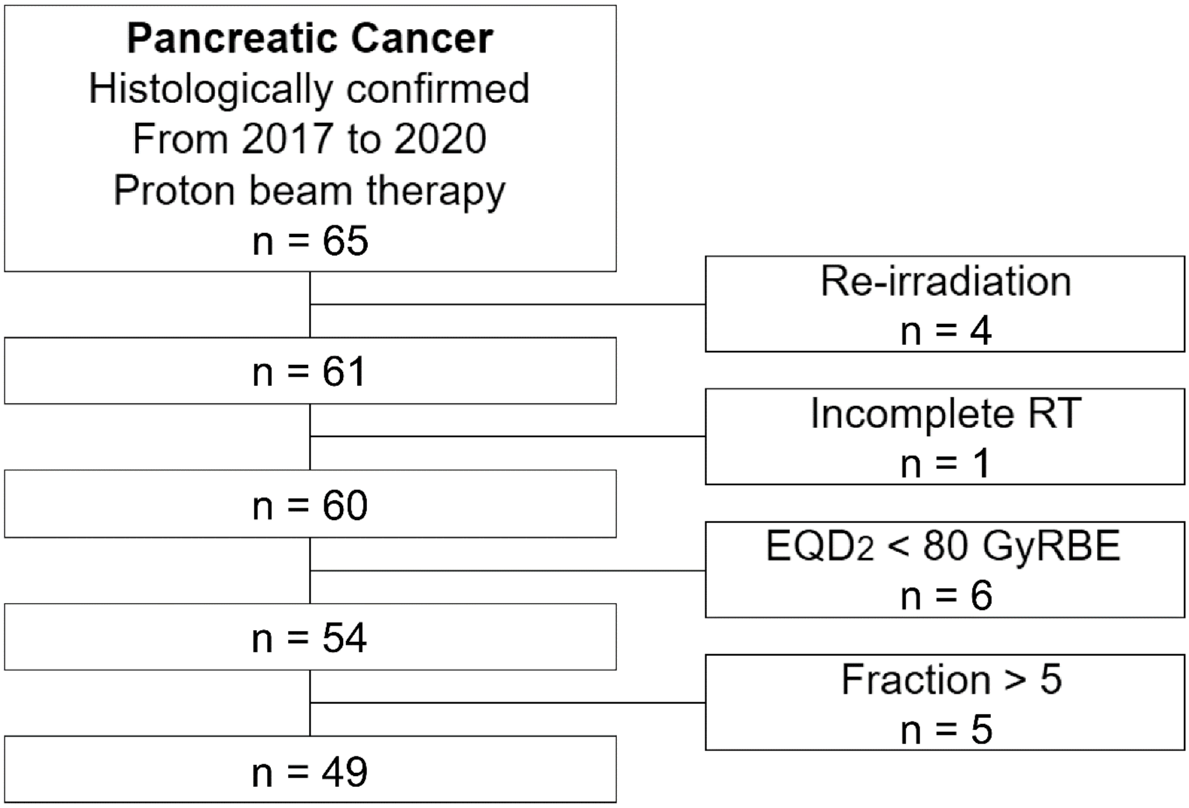

Figure S2. Kaplan–Meier survival curves of all patients treated with proton beam therapy (N = 65) (**A**) Overall survival (**B**) Progression-free survival (**C**) Local control rate.

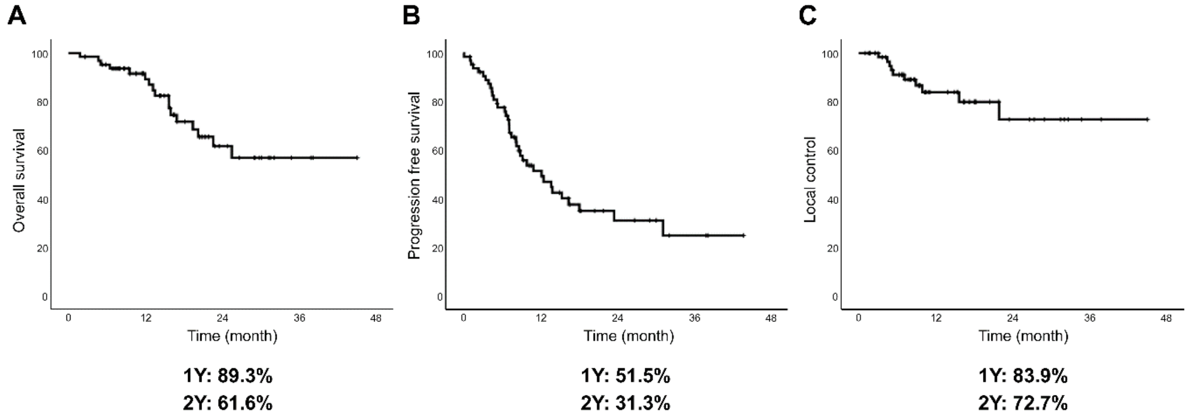

Figure S3. (**A**) Abdominal computed tomography (CT) of a patient who underwent primary repair of gastric perforation (**B**) Abdominal CT and endoscopic findings in a patient who underwent total gastrectomy.

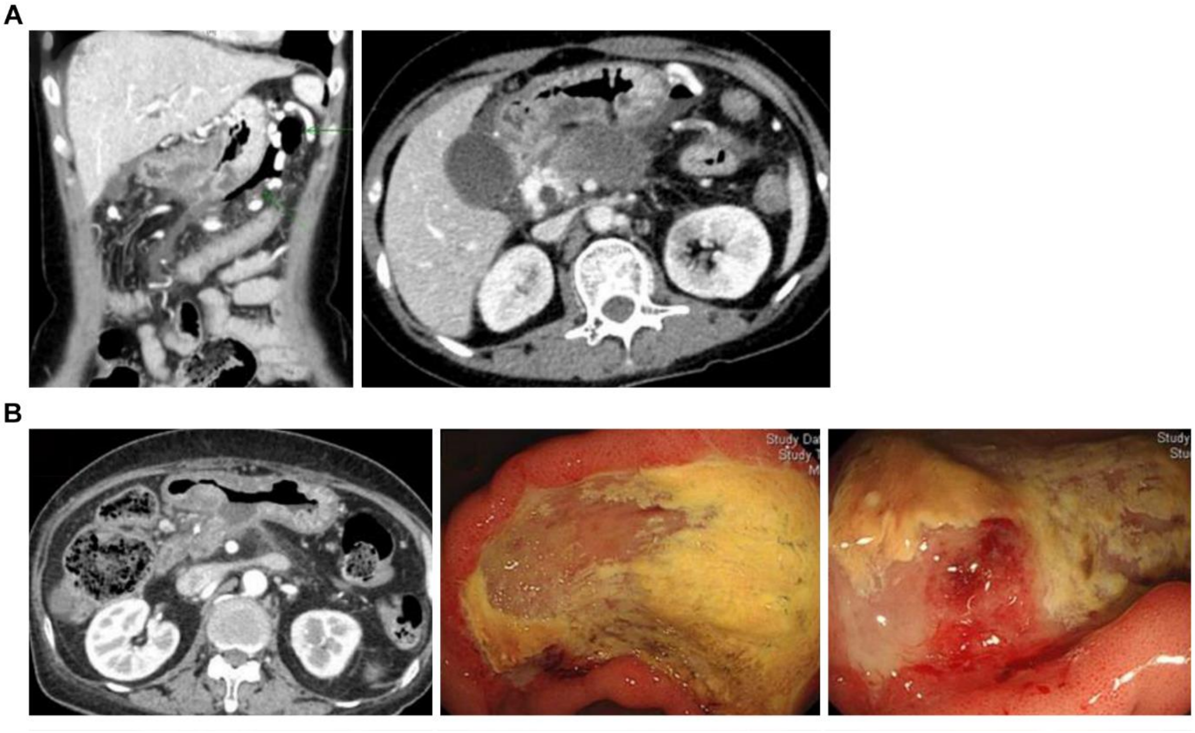

Table S1. Treatment-related gastroduodenal toxicities after stereotactic body proton beam therapy (n = 49).

Toxicity	Grade 1	Grade 2	Grade 3	Grade 4	Grade 5	Total
gastroduodenal ulcer	2 (4.1)	11 (22.4)	1 (2.0)	2 (4.1)	0	16 (32.7)
gastroduodenal perforation	0	0	0	1 (2.0)	0	1 (2.0)
gastroduodenal stricture	0	0	0	0	0	0

Values are presented as number (%).

SBPT: Stereotactic body proton beam therapy.
